# Comparing the effects of China’s three basic health insurance schemes on the equity of health-related quality of life: using the method of coarsened exact matching

**DOI:** 10.1186/s12955-018-0868-0

**Published:** 2018-03-07

**Authors:** Min Su, Zhongliang Zhou, Yafei Si, Xiaolin Wei, Yongjian Xu, Xiaojing Fan, Gang Chen

**Affiliations:** 1 School of Public Policy and Administration, Xi’an Jiaotong University, Xi’an, China; 2 Division of Clinical Public Health, and Institute of Health Policy Management and Evaluation, Dalla Lana School of Public Health, University of Toronto, Toronto, Canada; 3 School of Public Health, Health Science Center, Xi’an Jiaotong University, Xi’an, China; 4 Monash Business School, Monash University, Clayton, Australia

**Keywords:** China, Basic health insurance schemes, Health-related quality of life, Coarsened exact matching, Income-related health equity, Decomposition

## Abstract

**Background:**

China has three basic health insurance schemes: Urban Employee Basic Medical Insurance (UEBMI), Urban Resident Basic Medical Insurance (URBMI) and New Rural Cooperative Medical Scheme (NRCMS). This study aimed to compare the equity of health-related quality of life (HRQoL) of residents under any two of the schemes.

**Methods:**

Using data from the 5th National Health Services Survey of Shaanxi Province, China, coarsened exact matching method was employed to control confounding factors. We included a matched sample of 6802 respondents between UEBMI and URBMI, 34,169 respondents between UEBMI and NRCMS, and 36,928 respondents between URBMI and NRCMS. HRQoL was measured by EQ-5D-3L based on the Chinese-specific value set. Concentration index was adopted to assess health inequality and was decomposed into its contributing factors to explain health inequality.

**Results:**

After matching, the horizontal inequity indexes were 0.0036 and 0.0045 in UEBMI and URBMI, 0.0035 and 0.0058 in UEBMI and NRCMS, and 0.0053 and 0.0052 in URBMI and NRCMS respectively, which were mainly explained by age, educational and economic statuses. The findings demonstrated the pro-rich health inequity was much higher for the rural scheme than that for the urban ones.

**Conclusion:**

This study highlights the need to consolidate all three schemes by administrating uniformly, merging funds pooling and benefit packages. Based on the contributing factors, strategies aim to facilitate health conditions of the elderly, narrow economic gap, and reduce educational inequity, are essential. This study will provide evidence-based strategies on consolidating the fragmented health schemes towards reducing health inequity in both China and other developing countries.

## Background

Health equity has long been regarded as an overarching objective pursued by the whole health systems [1]. The evidence from previous research suggested that health insurance which designed to improve health care access could reduce health inequity [2, 3]. Therefore, China has implemented a series of comprehensive reforms to develop health insurance schemes [2, 4, 5]. There are three basic health insurance schemes in China. Urban Employee Basic Medical Insurance (UEBMI), a compulsory scheme, was established for the urban residents who work in the formal at the end of 1998. UEBMI is financed by payroll taxes from employers (6%) and employees (2%) [6, 7]. The inpatient care is covered by a pooled fund and outpatient care is supported by Individual Account [6, 7]. By the end of 2013, there were approximately 274.16 million people enrolled in the UEBMI, constituting 37.50% of the total urban residents. Urban Resident Basic Medical Insurance (URBMI), a voluntary scheme, was piloted in 2007 for the rest urban residents without formal jobs or unemployed such as children, students, elderly, and the young unemployed. URBMI has a Social Pooling Account for inpatient care and a Household Account for critical outpatient care (e.g. chronic or fatal disease). Financing is from the insured residents and local government. In 2013, the average per capital financing support of URBMI was RMB 380 (US$ 61.29), among which the insured resident was responsible for RMB 120(US$19.35) [8]. By the end of 2013, there were approximately 299.06 million people enrolled in the URBMI, accounting for 40.90% for the total urban people respectively. Additionally, New Rural Cooperative Medical Scheme (NRCMS), a voluntary scheme, was piloted in 2003 for the rural areas. NRCMS also has a Social Pooling Account and Household Account [9–11]. In 2013, the average per capital financing support of NRCMS was RMB 370 (US$ 59.68), among which the participant was responsible for RMB 90 (US$14.52) [8]. By the end of 2013, there were 0.80 billion people (accounting for 98.70% for the total rural people) enrolled in the NRCMS [8].

However, fragmentation in basic health insurance schemes is a significant factor for inequitable access to health care and financial protection for population covered by different insurance schemes in China [11]. Fragmentation means that there are a great number of separate funding mechanisms (e.g. many small insurance schemes) and a wide range of healthcare providers paid from different funding pools [12, 13]. In China, the administration, pooling levels and benefit packages are quite different across different schemes (Table 1), which result in the fragmented health insurance schemes. Specifically, NRCMS is administrated by the Chinese National Health and Family Planning Commission, while UEBMI and URBMI are administrated by the Chinese Ministry of Human Resources and Social Security. In terms of the funding pool, NRCMS is pooled at county level, while UEBMI and URBMI are pooled at municipal level. There are approximately 2856 counties and 333 municipalities in China, which indicates that there are about 2856 NRCMS schemes, 333 UEBMI schemes and 333 URBMI schemes in China. Third, reimbursement rate was 10% lower and healthcare coverage was much smaller for NRCMS than that for UEBMI and URBMI [11, 14, 15]. Fragmentation in basic health insurance schemes may have negative effects on health equity to some extent [11, 4]. Therefore, establishing a consolidated health insurance scheme by 2020 is one of the crucial objectives of the whole health system reforms in China (both vertical consolidation and horizontal consolidation). For vertical consolidation, the fund pooling and management of NCMS (from county level) and UEBMI and URBMI (from municipal level) should be moved to provincial and then country levels. For horizontal consolidation, the funding pools, management offices and information systems of three basic health insurance schemes should be merged together to reduce human resources costs [11–13]. Experience across the world demonstrates that consolidation of health insurance schemes has a multitude of benefits [6, 14, 15]. For example, consolidating the fragmented schemes, in theory, could extend financial pools, promote the efficiency in administration, narrow the inequities in benefit package and improve the access of health care, thereby reducing health inequity to some extent. So Chinese government has formulated a national guideline in 2016 to guide the consolidation of the existing fragmented schemes.

**Table 1:** Summary of basic health insurance schemes in China in 2013

	UEBMI	URBMI	NRCMS
Basic information			
Year of launch	1999	2007	2003
Target population	Urban employees in formal sectors	Urban residents without formal jobs or unemployed: children, students, elderly people	Rural residents
Enrolment type	Compulsory	Voluntary	Voluntary
Enrolment (n, %)	274.16million (37.50%)	299.06 million (40.90%)	0.80 billion (98.70%)
Administration	Chinese Ministry of Human Resources and Social Security	Chinese Ministry of Human Resources and Social Security	Chinese National Health and Family Planning Commission
Risk-pooling units	Municipal level, 2856 Risk-pooling units	Municipal level, 333 Risk-pooling units	County level, 333 Risk-pooling units
Benefit packages			
Service coverage	A Social Pooling Account for inpatient care and an Individual Account for outpatient care (generous)	A Social Pooling Account for inpatient care and a Household Account for critical outpatient care (i.e. chronic or fatal disease) (limited)	NRCMS included a Social Pooling Account for inpatient care and a Household Account for critical outpatient care (i.e. chronic or fatal disease) (limited)
Total premium per person	–	RMB 380(US$61.29)	RMB370(US$59.68)
Government subsidy per person	0	RMB 260(US$41.94)	RMB 280(US$45.16)
Individual contribution	2–3% of salary	RMB 120(US$19.35)	RMB 90(US$14.52)
Employer contribution	6–8% of salary	0	0
Actual reimbursement ratio (Inpatient care)	68.8%	53.62%	50.15%

Data resource: National Health Statistics Annual Report and Qingyue Meng et al., [11]

Recent decades have witnessed a wide range of research projects providing worthy insights into the effects of health insurance schemes on population health. The evidence showed that observational studies, natural experiments and experimental studies have been conducted to investigate the relationship between health insurance and health. Apart from this, both objective (e.g. mortality, birth weight, blood pressure) and subjective (e.g. self-reported health, health-related quality of life) health outcome measures have been used to explore the impacts of health insurance schemes on population health. Based on the literature review [16, 17], convincing evidence indicated that basic health insurance schemes can improve the health of vulnerable subgroups, such as elderly people, children and other individuals with specific health problems, especially those with low income. The related research in China reached similar conclusions. For example, an often-voiced opinion was that the health of the insured rural residents has been improved greatly since the establishment of NRCMS [11]. By using the EQ-5D instrument, Wang et al. [18] found the pilot of Rural Mutual Health Care (RMHC), which could be regarded as one type of the NRCMS, has led to decreases in pain or discomfort, and anxiety or depression for the insured, and shown positive impacts on mobility and usual activity for the elderly. Based on the URBMI survey conducted from 2007 to 2010, Pan et al. found URBMI was associated with better self-reported health [19, 20]. However, few evidence also indicated that the health condition of the insured was not improved significantly [21, 22]. In addition, few studies were available to compare the potential effects of different health insurance schemes on health equity.

This study aimed to fill the gap by comparing the income-related inequity in health-related quality of life (HRQoL) of the insured residents with different health insurances: between UEBMI and URBMI, UEBMI and NRCMS, and URBMI and NRCMS respectively. There are two key strengths in general. Firstly, we estimated the effects of the three basic health insurance schemes in China on the income-related inequity of HRQoL based on the coarsened exact matching (CEM) method to guarantee better balance of empirical distributions of the covariates between the comparison groups [23, 24]. Secondly, HRQoL, scored by the EQ-5D-3L instrument based upon the preferences of the Chinese general population, was employed as a health outcome measure [25]. Although, HRQoL has long been extensively applied in the research of health inequity in international literature across the world [26, 27], it was the first time to compare the effects of UEBMI, URBMI and NRCMS on the income-related inequity of HRQoL using the CEM method.

## Methods

### Data

Data were derived from the 5th National Health Services Survey (NHSS) of Shaanxi Province, Western China, in 2013. The NHSS has been initiated and conducted by the National Health and Family Planning Commission of China every 5 years since 1993 [28]. Shaanxi Province is situated in the northwest of China. There were roughly 37.60 million population living in the area with more than 205,800 km^2^, among which 48.70% of residents were living in rural areas. By the end of 2013, the per capital Gross Regional Product (GRP) was RMB 43,117 (US$ 6954, US$ were obtained by the annual average exchange rate US$1 = RMB 6.20 in 2013), which was lower than the per capita Gross Domestic product (GDP) of China (RMB 43,852, US$ 7072) [8].

By using a face-to-face interview combined with a standardized questionnaire which consisted of basic information concerning each respondent’s demographic as well as socio-economic characteristics, health conditions measured by the EQ-5D-3L instrument and self-reported illness and chronic disease; health insurance characteristics and health service utilizations. Additionally, there are three questions to get the information about health insurance characteristics: 1) Do you have health insurance; and 2) Which type of health insurance do you have; and 3) Do you buy any supplementary health insurance? By using the four-stage, stratified, random sampling method, 32 counties or districts of Shaanxi Province were selected randomly in the first stage, and 160 townships or streets were selected in the next stage. Thirdly, 320 villages or communities were chosen. Finally, 20,700 households (57,529 people) were interviewed. Since this study mainly focuses on the respondents aged 15 years and older, 5079, 2443 and 37,877 respondents covered by UEBMI, URBMI and NRCMS respectively were identified in the final sample for further analysis after data cleaning.

### Health-related quality of life

The EQ-5D-3L, with 245 kinds of health statuses, is the most widely used instrument to measure HRQoL based on people’s preferences across the world [29]. There are five dimensions (e.g. mobility, self-care, usual activities, pain or discomfort and anxiety or depression) and three response levels (e.g. no problems, some problems and extreme problems) for each dimension [30]. Over the past few decades, the utilization of the EQ-5D-3L was confined in China as the lack of Chinese value set. In our research, a tariff of Chinese people’s preferences based on the time trade-off method, which ranges from − 0.149 to 1 (− 0.149 stands for death and 0 stands for full health), was employed to measure the HRQoL [25].

### Coarsened exact matching method

It is more likely to neglect other potential confounding influences of different comparison groups that may result in health inequity [31] by roughly comparing the income-related inequity of HRQoL between different health insurance schemes. Therefore, we applied the coarsened exact matching (CEM) method, firstly proposed by Lacus, et al. [23, 24] to keep better balance of distributions of the covariates between the comparison groups and thereby reducing the biases. In general, the basic algorithm of CEM mainly includes three procedures. Firstly, each variable is coarsened by recoding to group and appoint the indistinguishable values with the same value. In the second step, the algorithm of exact matching is employed. And then removing the coarsened data, the final matched data should be reserved [23, 24]. A weighting variable generated by CEM method is used to equalize the number of observations within comparison groups [23, 24]. For balance checking, multivariate imbalance measure L_1_ was used and it was a relative magnitude depending on the data and the selected covariates [23, 24]. L_1_ ranges from 0 (perfect global balance) to 1 (maximal imbalance) and larger value represents larger imbalance between comparison groups. A good matching performance would bring a substantial reduction in L_1_ [23, 24]. L_1_ can be calculated as follows: Firstly, covariates were coarsened into bins. And then the discretized variables were cross-tabulated as X_1_ × …… × X_k_ for the treated and the control groups separately, and recorded the k-dimensional relative frequencies for the treated and ${f}_{\varepsilon_1\dots \dots {\varepsilon}_k}$for the control ${g}_{\varepsilon_1\dots \dots {\varepsilon}_k}$ units. Finally, the measure of imbalance is the absolute difference over all the cell values [24].

1 $$ {L}_{1\left(f,g\right)}=\frac{1}{2}\sum \limits_{\varepsilon_1\dots \dots {\varepsilon}_k}\mid {f}_{\varepsilon_1\dots \dots {\varepsilon}_k}-{g}_{\varepsilon_1\dots \dots {\varepsilon}_k}\mid $$

More details of the CEM method can be found in other literature [23, 24, 32, 33]. The CEM method was modeled by using the *cem* command code.

### Health inequity

#### Concentration index

Concentration index (CI) was applied to quantify the income-related inequality in HRQoL [34–36]. CI ranges from − 1 to 1, and 0 represents no income-related inequality of HRQoL [37, 38]. The positive score of CI shows pro-rich inequality of HRQoL and the negative score of CI shows pro-poor inequality in HRQoL. The Eq.2 was used to calculate the CI:

2 $$ \mathrm{C}=\frac{2}{\mu}\mathit{\operatorname{cov}}\left(y,r\right) $$

where C represents concentration index, *y * represents HRQoL, μ represents the mean of overall EQ-5D utility value, γ represents the fractional rank of income distribution [39, 40].

#### Decomposition methods of the CI

The income-related inequality in HRQoL could be explained by decomposing the CI into its contributing factors based on a regression, using Eqs. 3 and 4 [41].

3 $$ {y}_i=\alpha +\sum \limits_j{\beta}_j^m{x}_{ji}+\sum \limits_k{r}_k^n{z}_{ki}+{\varepsilon}_i $$

where *y*_*i*_ represents EQ-5D utility value; χ represents the unavoidable determinants of HRQoL (gender and age); z represents the avoidable determinants of HRQoL (marital states, educational states, commercial insurance and annual personal expenditure);${\beta}_j^m$ and${r}_k^n$are marginal effects of each variable;*εi * stands for the error. The decomposition of the concentration index C could be defined as:

4 $$ \mathrm{C}={\sum}_j\left(\raisebox{1ex}{${\beta}_j^m\overline{x_j}$}\!\left/ \!\raisebox{-1ex}{$\mu $}\right.\right){C}_j+\raisebox{1ex}{${GC}_{\varepsilon }$}\!\left/ \!\raisebox{-1ex}{$\mu $}\right. $$

Where μ is the mean of *y *, *C*_*j*_ are the CI for *xj * and $\overline{x_j}$are the means of *x*_*j*_. The contributions of independent variables are showed by the first item on the right side of Eq. 3 to the inequality of HRQoL, the last term is the generalized CI of ε [39, 40].

Tobit model was used to estimate Eq. 3 because the EQ-5D utility value is the limited dependent variable and usually has a ceiling effect (a great deal of residents had full health) [34]. The partial effects of independent variables were reported.

#### Horizontal inequity index

The horizontal inequity index (HI) of HRQoL was measured by deducting the contributions of unavoidable variables from the CI of HRQoL [42–44]. In this study, the HI of HRQoL was obtained by removing the contributions of unavoidable variables (such as gender and age) from the overall CI of HRQoL. A positive (negative) HI of HRQoL indicated the pro-rich (pro-poor) inequity. All analyses were performed in Stata version 13.0.

### Ethics

The ethics approval was obtained by the Ethics Committee of Xi’an Jiaotong University Health Science Center (approval number: 2015–644). Informed consent was obtained and the data was anonymized when analyzed.

## Results

### Matching performance

Table 2 showed the basic characteristics of the independent variables. It was obvious that there was significant difference on any social-demographic characteristic (except commercial medical insurance between UEBMI and URBMI) between UEBMI and URBMI, UEBMI and NRCMS and URBMI and NRCMS respectively.

**Table 2:** Baseline characteristics for the UEBMI, URBMI and NRCMS insured residents before matching

	UEBMI-URBMI			UEBMI-NRCMS			URBMI-NRCMS		
	UEBMI	URBMI	*p*-value	UEBMI	NRCMS	*p*-value	URBMI	NRCMS	*p*-value
Gender^b^									
Male^a^	2878(56.66)	14,679(60.05)	< 0.001	2878(56.66)	18,335(48.41)	< 0.001	1467(60.05)	18,335(48.41)	< 0.001
Female	2210(43.34)	976(39.95)		2210(43.34)	19,542(51.59)		976 (39.95)	19,542(51.59)	
Age(years)^b^									
15–44^a^	1541(30.34)	1360(55.67)	< 0.001	1541(30.34)	16,343(43.15)	< 0.001	1360(55.67)	16,343(43.15)	< 0.001
45–59	1574(30.99)	5459(22.31)		1574(30.99	12,679(33.47)		545(22.31)	12,679(33.47)	
> 60	1964(38.67)	538(22.02)		1964(38.67)	8855(23.38)		538(22.02)	8855(23.38)	
Marital status^b^									
Single^a^	219(4.31)	542(22.21)	< 0.001	219 (4.31)	4796(12.68)	< 0.001	542(22.21)	4796(12.68)	< 0.001
Marriage	4507(88.79)	1680(68.85)		4507(88.79)	29,857(78.94)		1680 (68.85)	29,857(78.94)	
Widowed	350(6.90)	218(8.93)		350(6.90)	3171(8.38)		218(8.93)	3171(8.38)	
Education status^b^									
illiterate^a^	105(2.07)	219(8.89)	< 0.001	105(2.07)	6700(17.69)	< 0.001	219(8.89)	6700(17.69)	< 0.001
Primary school	402(7.91)	283(11.59)		402(7.91)	10,248(27.06)		283(11.59)	10,248(27.06)	
Junior middle school	1235(24.32)	763(31.24)		1235(24.32)	15,588(41.16)		763(31.24)	15,588(41.16)	
Senior middle school	1803(35.50)	778(31.86)		1803(35.50)	4347(11.48)		778(31.86)	4347(1.48)	
College degree or above	1534(30.20)	399(16.34)		1534(30.20)	987(2.61)		399(16.34)	987(2.61)	
Commercial insurance^b^									
Yes	257(5.06)	125(5.12)	0.917	257(5.06)	1501(3.96)	< 0.001	125(5.12)	1501(3.96)	0.005
No^a^	4822(94.94)	2318(94.88)		4822(94.94)	36,376(96.04)		2318 (94.88)	36,376(96.04)	
Annual personal expenditure^c^									
Poorest^a^	3394.39(929.31)	3142.58(1106.71)	< 0.001	1652.39(510.40)	1515.80(510.27)	< 0.001	1572.44(443.31)	1465.75(488.55)	< 0.001
2nd	5750.21(593.41)	5728.38(526.94)		2981.93(362.40)	2910.66(365.06)		2812.79(313.97)	2799.84(349.40)	
Middle	7780.67(635.76)	7772.94(618.55)		4395.03(451.18)	4286.20(453.74)		4107.09(424.03)	4077.30(410.67)	
4th	10,498.06(1045.30)	10,490.97(1108.87)		6476.46(758.45)	6248.56(756.70)		5985.61(669.48)	5852.68(667.65)	
Richest	19,615.01(9039.77)	19,365.29(7738.98)		13,895.05(7621.64)	11,869.14(5830.35)		12,583.80(6438.38)	11,177.45(5510.76)	
N	5079	2443		5079	37,877		2443	37,877	

Mean (SD) or N (%) are reported

Statistical test is used for balance checking between different health insurance schemes before matching. It was obvious that there was significant difference on any social-demographic characteristic (except commercial medical insurance between UEBMI and URBMI) between UEBMI and URBMI, UEBMI and NRCMS and URBMI and NRCMS respectively

*UEBMI* Urban Employee Basic Medical Insurance, *URBMI* Urban Resident Basic Medical Insurance, *NRCMS* New Rural Cooperative Medical Scheme

^a^Reference levels in the Tobit regression

^b^Chi-square test

^c^Students’ T-test

The multivariate L_1_ statistics were reported in Table 3. After matching, L_1_ between UEBMI and URBMI, UEBMI and NRCMS and URBMI and NRCMS were all actually close to zero, which were much lower than that before matching (0.507, 0.709 and 0.533 respectively). Table 4 showed there was no statistical difference on any characteristic between UEBMI and URBMI, UEBMI and NRCMS and URBMI and NRCMS (*P* > 0.10), which indicated good matching performances and thus different groups became more comparable.

**Table 3:** The L_1_ measure of imbalance before and after Coarsened Exact Matching

	UEBMI-URBMI		UEBMI-NRCMS		URBMI-NRCMS	
	Before Matching	After Matching	Before Matching	After Matching	Before Matching	After Matching
	L_1_(mean)	L_1_(mean)	L_1_(mean)	L_1_(mean)	L_1_(mean)	L_1_(mean)
Gender	0.167 (0.167)	2.0e-15 (−1.4e-15)	0.083 (0.083)	1.6e-14 (−1.0e-14)	0.084 (− 0.084)	3.4e-14 (1.7e-14)
Age	0.253 (0.419)	1.1e-15 (−6.2e-15)	0.153 (0.280)	1.4e-14 (−1.1e-13)	0.125 (− 0.139)	3.4e-14 (−9.1e-14)
Marital status	0.199 (0.169)	2.5e-16 (−2.7e-15)	0.099 (0.069)	5.3e-15 (−7.1e-14)	0.101 (−0.091)	1.1e-14 (−6.5e-14)
Education status	0.175 (0.488)	7.7e-16 (−2.7e-15)	0.516 (1.296)	1.2e-14 (3.1e-15)	0.341 (0.808)	2.6e-14 (5.5e-14)
Commercial insurance	0.001 (−0.001)	3.5e-16 (−2.4e-17)	0.011 (0.011)	1.4e-15 (−1.1e-15)	0.012 (0.012)	7.9e-15 (−3.1e-15)
Annual personal expenditure	0.139 (0.465)	6.4e-16 (−1.3e-15)	0.451 (1.395)	1.2e-14 (−2.0e-13)	0.331 (1.041)	3.5e-14 (−1.4e-13)
Multivariate L_1_	0.507	2.751e-16	0.709	9.850e-15	0.533	3.086e-14
N	7522	6802	42,956	34,169	40,320	36,928

L_1_ reported the L_1j_ measure, which is L_1_ computed for the j_th_ variable separated. The mean was labeled in parentheses reported the difference in means

**Table 4:** Baseline characteristics for the UEBMI, URBMI and NRCMS insured residents after matching

	UEBMI-URBMI			UEBMI-NRCMS			URBMI-NRCMS		
	UEBMI	URBMI	*P*-value^*^	UEBMI	NRCMS	*P*-value^*^	URBMI	NRCMS	*P*-value^*^
Gender^b^									
Male^a^	2578(56.56)	9749(56.56)	0.994	2789 (57.22)	16,763(57.22)	1.000	969(39.94)	13,795(39.04)	1.000
Female	1980(43.44)	1269(43.44)		2085 (42.78)	12,531(42.78)		1456(60.06)	20,742(60.06)	
Age(years)^b^									
15–44^a^	1512(33.17)	744 (33.17)	1.000	1531(31.41)	9202(31.41)	1.000	1357(55.94)	19,319(55.94)	1.000
45–59	1432(31.42)	705(31.42)		1534(31.47)	9220(31.47)		541(22.30)	7702(22.30)	
> 60	1614(35.41)	794(35.41)		1809(37.12)	10,872(37.12)		528(21.76)	7516(21.76)	
Marital status^b^									
Single^a^	189(4.15)	93(4.15)	1.000	209(4.29)	1256(4.29)	1.000	540(22.26)	7687(22.26)	1.000
Marriage	4166(91.40)	2051(91.40)		4348(89.21)	26,133(89.21)		1670(68.84)	23,775(68.84)	
Others	203(4.45)	99(4.45)		317(6.50)	1905(6.50)		216(8.90)	3075(8.90)	
Education status^b^									
illiterate^a^	85(1.86)	419(1.86)	1.000	105(2.15)	631(2.15)	1.000	218(8.99)	3103(8.99)	1.000
Primary school	366(8.03)	180(8.03)		400(8.21)	2404 (8.21)		282(11.62)	4014(11.62)	
Junior middle school	1163(25.52)	5729(25.52)		1229(25.22)	7386(25.22)		760(31.33)	10,819(31.33)	
senior middle school	1691(37.10)	832(37.10)		1781(36.54)	10,704(36.54)		777(32.03)	11,061(32.03)	
College degree or above	1253(27.49)	616(27.49)		1359(27.88)	8168(27.88)		389(16.03)	5538(16.03)	
Commercial insurance^b^									
Yes	133(2.92)	65(2.92)	1.000	216(4.43)	1298(4.43)	1.000	121(4.99)	1722(4.99)	1.000
No^a^	4425(97.08)	2178(97.08)		46,589(95.57)	27,996(95.57)		2305(95.01)	32,815(95.01)	
Annual personal expenditure^c^									
Poorest^a^	3392.11(921.68)	3113.49(1102.49)	0.690	1659.30(509.97)	1546.73(493.41)	1.000	1563.43(444.63)	1464.44(486.55)	0.998
2nd	5747.30(593.99)	5682.16(555.48)		2983.28(363.14)	2909.50(356.08)		2806.92(313.97)	2808.21(346.39)	
Middle	7773.45(634.24)	7737.22(606.65)		4391.29(451.32)	4287.36(455.65)		4094.72(424.60)	4065.54(411.58)	
4th	10,495.69(1047.65)	10,492.19(1132.61)		6480.34(762.07)	6247.63(751.97)		5998.79(668.40)	5869.55(671.31)	
Richest	19,622 .00(9026.64)	19,508.92(9674.81)		13,887.19(7566.93)	12,399.50(5793.02)		12,509.00(6442.53)	11,549.90(5445.48)	
N	4558	2244		4874	29,295		2423	34,505	

Mean (SD) or N (%) are reported

There was no statistical difference on any characteristic between UEBMI and URBMI, UEBMI and NRCMS and URBMI and NRCMS (*P* > 0.10)

P^*^ Considering matching weights

^a^Reference levels in the Tobit regression

^b^Chi-square test

^c^Students’ T-test

Three new databases generated by the CEM method were applied to study the health equity. The first database contained 4558 UEBMI insured residents and 2244 URBMI enrollees, the second one contained 4874 UEBMI insured residents and 29,295 NRCMS enrollees, whilst the third one contained 2426 URBMI insured residents and 34,538 NRCMS enrollees.

### Description of EQ-5D dimensions

The utility value of each dimension and the mean utility value were presented in Table 5. The results of the matched sample showed the mean utility value were 0.9589 [95% CI (0.9553, 0.9626)] and 0.9449 [95% CI (0.9387, 0.9511)] in UEBMI and URBMI, 0.9579 [95% CI (0.9543, 0.9614)] and 0.9473 [95% CI (0.9457, 0.9488)] in UEBMI and NRCMS and 0.9505 [95% CI (0.9450, 0.9559)] and 0.9605 [95% CI (0.9592, 0.9619)] in URBMI and NRCMS respectively. The differences of mean utility scores between UEBMI and URBMI, UEBMI and NRCMS and URBMI and NRCMS were all significant.

**Table 5:** Mean and 95% confidence interval (CI) of the EQ-5D scores

	UEBMI-URBMI		UEBMI-NRCMS		URBMI-NRCMS	
	UEBMI	URBMI	UEBMI	NRCMS	URBMI	NRCMS
	Mean (95% CI)	Mean (95% CI)	Mean (95% CI)	Mean (95% CI)	Mean (95% CI)	Mean (95% CI)
Mobility	− 0.0074 (− 0.0083, − 0.0065)	− 0.0082 (− 0.0095, − 0.0068)	− 0.0076 (− 0.0085, − 0.0067)	− 0.0089 (− 0.0082, − 0.0085)	− 0.0084 (− 0.0096, − 0.0072)	− 0.0064 (− 0.0067, − 0.0061)
Selfcare	− 0.0046 (− 0.0053, − 0.0038)	− 0.0055 (− 0.0066, − 0.0043)	− 0.0047 (− 0.0054, − 0.0040)	− 0.0056 (− 0.0059, − 0.0053)	− 0.0053 (− 0.0064, − 0.0043)	− 0.0042 (− 0.0045, − 0.0040)
Activity	− 0.0051 (− 0.0059, − 0.0044)	− 0.0082 (− 0.0096, − 0.0068)	− 0.0053 (− 0.0061, − 0.0046)	− 0.0067 (− 0.0071, − 0.0064)	− 0.0067 (− 0.0078, − 0.0055)	− 0.0051 (− 0.0053, − 0.0048)
Pain	− 0.0120 (− 0.0130, − 0.0110)	− 0.0142 (− 0.0157, − 0.0125)	− 0.0123 (− 0.0133, − 0.0113)	− 0.0158 (− 0.0163, − 0.0154)	− 0.0138 (− 0.0153, − 0.0123)	− 0.0117 (− 0.0120, − 0.0113)
Anxiety	− 0.0053 (− 0.0060, − 0.0047)	− 0.0107 (− 0.0122, − 0.0093)	− 0.0054 (− 0.0060, − 0.0047)	− 0.0077 (− 0.0080, − 0.0074)	− 0.0080 (− 0.0091, − 0.0067)	− 0.0062 (− 0.0064, − 0.0059)
EQ5D	0.9589 (0.9553, 0.9626)	0.9449 (0.9387, 0.9511)	0.9579 (0.9543, 0.9614)	0.9473 (0.9457, 0.9488)	0.9505 (0.9450, 0.9559)	0.9605 (0.9592, 0.9619)

Differences of mean EQ5D utilities across the three basic health insurances were significant based on the Mann-Whitney U test; The difference across URBMI and NRCMS was not statistically significant

### Concentration index and decomposition

#### Concentration index

Table 6 showed CIs of the 5 dimensions were all negative but the CIs of mean EQ-5D utility scores were all positive, suggesting that health issues were relatively significant among the poorer groups, however, the relatively higher EQ-5D utility scores were relatively significant among the richer groups. In other words, there tended to be less health problems and better HRQoL for the rich insured respondents than that of the poor counterparts covered by UEBMI, URBMI and NRCMS in Shaanxi province [45]. The CIs of overall utility scores were 0.0087 [95% CI (0.0065, 0.0110)] and 0.0083 [95% CI (0.0050, 0.0118)] in UEBMI and URBMI, 0.0074 [95% CI (0.0053, 0.0096)] and 0.0117 [95% CI (0.0108, 0.0127)] in UEBMI and NRCMS and 0.0084 [95% CI (0.0076, 0.0092)] and 0.0107 [95% CI (0.0099, 0.0116)] in URBMI and NRCMS respectively.

**Table 6:** Concentration index of the scores of EQ-5D and each of its dimension

	UEBMI-URBMI		UEBMI-NRCMS		URBMI-NRCMS	
	UEBMI	URBMI	UEBMI	NRCMS	URBMI	NRCMS
	Mean (95% CI)	Mean (95% CI)	Mean (95% CI)	Mean (95% CI)	Mean (95% CI)	Mean (95% CI)
Mobility	− 0.2758^***^ (− 0.3448, − 0.2067)	− 0.3006^***^ (− 0.3954, − 0.2058)	− 0.2474^***^ (− 0.3137, − 0.1811)	− 0.1989^***^ (− 0.2209, − 0.1769)	− 0.2301^***^ (− 0.0336, − 0.1466)	− 0.2316^***^ (− 0.2548, − 0.2084)
Selfcare	−0.3078^***^ (− 0.4011, − 0.2144)	−0.2532^***^ (− 0.3722, − 0.1342)	−0.2614^***^ (− 0.3504, − 0.1725)	−0.2318^***^ (− 0.2622, − 0.2013)	−0.1942^**^ (− 0.3091, − 0.0079)	−0.2611^***^ (− 0.2910, − 0.2312)
Activity	−0.2989^***^ (− 0.3822, − 0.2155)	−0.0469 (− 0.1446, − 0.0507)	−0.2448^***^ (− 0.3233, − 0.1663)	−0.1663^***^ (− 0.1908, − 0.1418)	−0.2070^***^ (− 0.3050, − 0.1091)	−0.2259^***^ (− 0.2516, − 0.2001)
Pain	−0.1450^***^ (− 0.1942, − 0.0956)	−0.0812^**^ (− 0.1439, − 0.0185)	−0.1104^***^ (− 0.1571, − 0.0638)	−0.0463^***^ (− 0.0593, − 0.0334)	−0.1288^***^ (− 0.1917, 0.0659)	−0.1559^***^ (− 0.1714, − 0.1403)
Anxiety	−0.1137^**^ (− 0.1868, − 0.0406)	−0.0112 (− 0.0891, 0.0668)	−0.0921^**^ (− 0.1619, − 0.0224)	0.0482^***^ (0.0290, 0.0675)	−0.1678^***^ (− 0.2490, − 0.0865)	−0.1274^***^ (− 0.1487, − 0.1060)
EQ5D	0.0087^***^ (0.0065, 0.0110)	0.0083^***^ (0.0050, 0.0118)	0.0074^***^ (0.0053, 0.0096)	0.0117^***^ (0.0108, 0.0127)	0.0084^***^ (0.0076, 0.0092)	0.0107^***^ (0.0099, 0.0116)

^*^P < 0.05;^**^P < 0.01;^***^P < 0.001;Differences of Concentration indexes of EQ5D utilities across UEBMI and NRCMS and URBMI and NRCMS were significant based on the Students’ T-test; Difference across UEBMI and URBMI was not significant

#### Decomposition of inequity of HRQoL

Table 7 presented the decomposition of CIs of the overall utility scores. The estimated partial effects of each related factor of health inequality was calculated based upon the Tobit model. Contribution to the inequality of the overall utility score and the proportion of contribution in the overall CIs were reported.

**Table 7:** Decomposition of concentration index for UEBMI, URBMI and NRCMS

	UEBMI- URBMI				UEBMI-NRCMS				URBMI-NRCMS			
	UEBMI		URBMI		UEBMI		NRCMS		URBMI		NRCMS	
	dy/dx	con (%)	dy/dx	con (%)	dy/dx	con (%)	dy/dx	con (%)	dy/dx	con (%)	dy/dx	con (%)
Gender(Female)	−0.0019	0.0000 (0.53)	−0.0052	0.0000 (0.41)	−0.0028	0.0001 (0.74)	0.0055^***^	−0.0001 (− 0.71)	−0.0061	0.0000 (0.29)	0.0037^**^	0.0000 (−0.07)
45–59	− 0.0168^***^	−0.0003 (− 3.63)	−0.0226^***^	0.0004 (5.16)	−0.0150^**^	− 0.0003 (− 3.70)	−0.0207^***^	− 0.0002 (− 1.94)	−0.0204^**^	0.0004 (5.07)	−0.0214^***^	0.0001 (0.48)
60 or above	−0.0607^***^	0.0051 (59.06)	−0.0662^***^	0.0038 (45.49)	−0.0570^***^	0.0041 (55.85)	−0.0690^***^	0.0060 (51.50)	−0.0576^***^	0.0028 (33.88)	−0.0765^***^	0.0053 (49.47)
Marriage	0.0059	0.0001 (0.96)	−0.0053	0.0000 (0.47)	0.0039	0.0001 (0.70)	−0.0044	0.0000 (0.02)	−0.0021	0.0000 (0.06)	−0.0009	0.0000 (−0.03)
Others	−0.0309^**^	0.0006 (6.72)	−0.0515^***^	0.0014 (16.64)	−0.0230^*^	0.0004 (5.19)	−0.0606^***^	0.0009 (7.93)	−0.0425^***^	0.0011 (13.53)	−0.0570^***^	0.0010 (9.05)
Primary school	0.0349^**^	−0.0009 (−10.85)	0.0502^***^	−0.0013 (− 16.22)	0.0527^***^	−0.0013 (− 17.12)	0.0448^***^	−0.0016 (− 13.81)	0.0666^***^	−0.0015 (− 17.40)	0.0374^***^	−0.0006 (− 5.49)
Junior middle school	0.0497^***^	−0.0021 (− 24.65)	0.0772^***^	− 0.0020 (− 23.61)	0.0735^***^	− 0.0034 (− 45.65)	0.0544^***^	0.0018 (15.33)	0.0967^***^	−0.0018 (− 21.12)	0.0487^***^	0.0013 (12.36)
Senior middle school	0.0506^***^	−0.0002 (− 2.50)	0.0814 ^***^	0.0031 (36.91)	0.0748^***^	−0.0009 (− 12.18)	0.0550^***^	0.0011 (9.57)	0.1043^***^	0.0031 (37.30)	0.0494^***^	0.0010 (9.76)
College degree or above	0.0565^***^	0.0048 (54.72)	0.0928^***^	0.0038 (45.43)	0.0827^***^	0.0070 (94.83)	0.0592^***^	0.0007 (5.69)	0.1166	0.0045 (54.09)	0.0532^***^	0.0005 (5.01)
Commercial insurance	0.0050	0.0001 (0.59)	−0.0034	0.0001 (1.25)	0.0105	0.0001 (1.30)	0.0066^***^	0.0001 (0.46)	0.0096	0.0002 (2.33)	0.0009^***^	0.0000 (0.10)
2nd	0.0015	−0.0001 (−1.64)	0.0034^***^	−0.0002 (−2.27)	0.0181	− 0.0011 (− 15.04)	0.0217^***^	−0.0022 (− 18.62)	0.0081^***^	−0.0007 (−8.11)	0.0170^***^	− 0.0016 (− 14.63)
Middle	0.0044	− 0.0001 (− 0.65)	0.0049	0.0002 (2.27)	0.0197	− 0.0017 (− 23.32)	0.0215^***^	0.0000 (− 0.01)	−0.0020	0.0000 (0.00)	0.0168^***^	0.0000 (−0.07)
4th	0.0091^***^	0.0007 (7.85)	−0.0016^***^	−0.0001 (− 1.73)	0.0227^*^	− 0.0020 (− 27.13)	0.0172^***^	0.0016 (13.87)	0.0039^***^	0.0003 (3.90)	0.0127^***^	0.0011 (10.40)
Richest	0.0051^***^	0.0009 (10.68)	−0.0034	−0.0005 (−5.49)	0.0244^*^	0.0063 (85.27)	0.0217^***^	0.0034 (29.29)	−0.0006	−0.0001 (−1.13)	0.0143^***^	0.0023 (21.87)
CI	0.0087		0.0083		0.0074		0.0117		0.0084		0.0107	
Con of health need	0.0049		0.0042		0.0039		0.0057		0.0033		0.0053	
HI	0.0036		0.0045		0.0035		0.0058		0.0053		0.0052	

dy/dx, the partial effect in Tobit regression model; Con, the contribution of regression to inequality of EQ-5D utility score; %, the share of contribution index of EQ-5D utility score

A positive marginal effect, such as education in UEBMI and URBMI indicated a higher educational level was significantly associated with better HRQoL, whilst a negative marginal effect, such as age, suggested the overall HRQoL decreased along the ageing population.

A positive (negative) contribution represented the variable raised (reduced) the pro-rich inequality (Liu et al., 2014). It can be seen immediately that majority of the HRQoL inequality were attributable to age, educational and economic statuses by defining the contributions as a proportion of each variable. Take the UEBMI and NRCMS for example, for the insured residents of UEBMI, we found that age, educational and economic statuses had the largest (52.15%), second largest (19.88%) and third largest (19.78%) contributions to explain the inequality of HRQoL. For the insured residents of NRCMS, age, economic and educational statuses had the largest (49.56%), second largest (24.53%) and third largest (16.78%) contributions to explain the inequality of HRQoL.

#### Horizontal inequity index

Table 7 showed the HI of EQ-5D utility score were 0.0036 and 0.0045 in UEBMI and URBMI, 0.0035 and 0.0058 in UEBMI and NRCMS and 0.0053 and 0.0052 in URBMI and NRCMS respectively.

## Discussions

We compared the equity of HRQoL of residents under any two of the schemes. The findings revealed that the insured residents of UEBMI reported higher HRQoL than that of URBMI and NRCMS. Furthermore, URBMI and NRCMS had higher pro-rich health inequity than that of UEBMI. Age, educational and economic statuses were key factors to explain the poor-rich inequity in HRQoL.

The first aim was to compare the effects of UEBMI, URBMI and NRCMS on HRQoL. The descriptive results of the mean EQ-5D utility score significantly suggested that compared with the UEBMI, the insured residents of URBMI and NRCMS had worse HRQoL, and health outcomes for the URBMI insured were the worst. To some extent, health service utilization could be one of the potential reasons, as the health status may be improved upon utilization. In Shaanxi Province, the annual hospitalization rates of UEBMI, NRCMS and URBMI were 14.66%, 9.95% and 9.56% respectively, and the outpatient visit rations within 2 weeks of UEBMI, NRCMS and URBMI were 12.12%, 12.89% and 11.67% respectively in 2013. Moreover, the percentages of non-hospitalized who should have been hospitalized of UEBMI, NRCMS and URBMI were 19.15%, 19.09% and 26.79% respectively and the percentages of non-visited who should have been visited of UEBMI, NRCMS and URBMI were 17.47%, 22.79% and 24.62% respectively.

The second aim was to compare the effects of UEBMI, URBMI and NRCMS on the equity of HRQoL. Based on the CEM matched samples, the positive CIs revealed that the rich people reported better HRQoL than the poor, which was consistent with most other previous research [46]. Moreover, comparing the CIs among three basic health insurance schemes, the results showed the pro-rich health inequality was much higher for the rural scheme than that for the urban ones. Since few related studies compared the effects of different health insurance schemes on the inequality of HRQoL, we could not compare this finding with other previous literature. However, theoretically, the goal of developing the health insurance was to protect households from financial catastrophe, and further improve the health service utilization, thus promoting health status by the benefit package design. Therefore, the differences of health inequality between rural and urban insured residents seemed alarming. To some extent, the fragmented benefit package designs could be responsible for the health inequalities among the UEBMI, URBMI and NRCMS. Take the benefit package designs of UEBMI and NRCMS for example. Firstly, UEBMI stipulated higher financing support than NRCMS. The insured residents of UEBMI is financed both by employers and employees, of which 6% of total salaries was supported by the employer and 2% of individual salary was financed by the employee. Whilst the average per capital financing support of NRCMS was RMB 365 (US$ 58.87) in 2013 (CNBS, 2014). Secondly, UEBMI stipulated higher actual reimbursement ratio than that of NRCMS (64.84% and 45.27% respectively in 2013) [8]. Thirdly, UEBMI included a Social Pooling Account for inpatient care and an Individual Account for outpatient care, whilst NRCMS included a Social Pooling Account for inpatient care and a Household Account for critical outpatient care (e.g. chronic or fatal disease) [36]. All these fragmented benefit package designs may result in the different pro-rich income-related health inequality among different schemes.

The third aim was to compare the HIs of UEBMI, URBMI and NRCMS. The positives HIs revealed that controlling the unavoidable characteristics of HRQoL, the URBMI had marginally higher pro-rich health inequity of HRQoL than that of NRCMS, and both of them had a higher pro-rich health inequity than that of UEBMI. Findings from the decomposition of inequality in HRQoL indicated the HIs were mainly explained by age, educational and economic statuses for both urban and rural insured residents, which was consistent with previous studies analyzing health inequity of the whole population of Shaanxi Province [36]. Therefore, we must consider the contributions of key determinants when formulating health policy interventions, allocating health resources and relieving health inequities. It is crucial to facilitate the health conditions of the aged population and narrow economic and educational gaps between the rich and the poor for both rural and urban insured residents.

There are some limitations should be noted. Firstly, owing to the cross-sectional data, only the association other than the causal inference between basic health insurance schemes and HRQoL was investigated. Secondly, the survey data was drawn from Shaanxi Province. Next, because of the data availability, we could not consider all the unobservable heterogeneities (e.g. URBMI and NRCMS are both voluntary schemes, but UEBMI is a compulsory counterpart) and other mediating factors such as access to healthy food, lifestyle and so forth, which may lead to bias. Additionally, the self-reported information on EQ-5D and healthcare utilization may be subjected to recall bias. Finally, employing the EQ-5D-3 L instrument to measure HRQoL also has some limitations. There exists a severe ceiling effect on EQ-5D, especially for the general population [46, 47]. Additionally, a related research indicated that the EQ-5D-3L instrument is not as sensitive as the SF-6D instrument in assessing HRQoL [40]. However, EQ-5D-3L is the only instrument that has been used in such large scale household surveys in China.

## Conclusions

This study has demonstrated the more generous health insurance scheme was generally associated with improved population heath and can reduce health inequity, particularly for the vulnerable groups. Furthermore, the income-related health inequities in HRQoL were mainly explained by age, educational and economic statuses for both rural and urban insured residents. Overall, it can be seen immediately that the basic health insurance schemes had positive impacts on decreasing the inequity in population health, but the effects were relatively limited, especially URBMI and NRCMS. Therefore, our results highlight the need to consolidate all three schemes and achieve the universal health coverage by administrating uniformly, merging funds pooling and consolidating the fragmented benefit packages and so on. Besides, based on the contributing factors which may result in health inequity, more efforts are urged to improve the health condition of the aged population, especially by preventing chronic diseases like hypertension and diabetes. Last, narrowing the economic gap by taking targeted measures in poverty alleviation and relieving educational inequity between the rich and the poor is helpful for improving HRQoL and then reducing pro-rich health inequity in Shaanxi Province.

## Abbreviations

CEM: Coarsened exact matching
CI: Concentration Index
EQ-5D: EuroQol 5 dimension
HI: Horizontal inequity index
HRQoL: Health-related quality of life
NHSS: National Health Services Survey
NRCMS: New Rural Cooperative Medical Scheme
RMHC: Rural Mutual Health Care
UEBMI: Urban Employee Basic Medical Insurance
URBMI: Urban Resident Basic Medical Insurance
